# High temperature boosts resistant starch content by altering starch structure and lipid content in rice *ssIIIa* mutants

**DOI:** 10.3389/fpls.2022.1059749

**Published:** 2022-11-18

**Authors:** Yufeng Zhou, Zhenfeng Cheng, Shuo Jiang, Jinxi Cen, Dianxing Wu, Xiaoli Shu

**Affiliations:** ^1^ State Key Laboratory of Rice Biology and Key Lab of the Ministry of Agriculture for Nuclear Agricultural Sciences, Institute of Nuclear Agricultural Sciences, Zhejiang University, Hangzhou, China; ^2^ Hainan Institute, Zhejiang University, Yazhou Bay Science and Technology City, Sanya, China

**Keywords:** resistant starch, high temperature, amylopectin, chain length distribution, rice

## Abstract

High temperature (HT) during grain filling had adverse influences on starch synthesis. In this study, the influences of HT on resistant starch (RS) formation in rice were investigated. Most genes in *ssIIIa* mutants especially in RS4 were upregulated under Normal Temperature (NT) while downregulated under HT when compared with those of wild parent R7954. *ssIIIa* mutants had higher RS content, more lipid accumulation, higher proportion of short chains of DP 9–15, and less long chains of DP ≥37. *ssIIIa* mutation exacerbated the influences of HT on starch metabolite and caused larger declines in the expression of *BEI*, *BEIIa*, *BEIIb*, and *SSIVb* when exposed to HT. HT reduced the contents of total starch and apparent amylose significantly in wild type but not in mutants. Meanwhile, lipids were enriched in all varieties, but the amounts of starch–lipid complexes and the RS content were only heightened in mutants under HT. HT led to greatest declines in the amount of DP 9–15 and increases in the proportion of fb3 (DP ≥37); the declines and increases were all larger in mutants, which resulted in varied starch crystallinity. The increased long-chain amylopectin and lipids may be the major contributor for the elevated RS content in mutants under HT through forming more starch–lipid complexes (RSV).

## Introduction

Rice (*Oryza sativa*) is an extremely important cereal and is the main food for more than 50% of the population worldwide. As the major components in rice grains, starch mainly comprises amylose and amylopectin molecules. The ratio of amylose to amylopectin and the structure of starch granules are key factors influencing rice quality. Meanwhile, amylose content and the distributions of branch lengths in both amylose and amylopectin influence starch digestibility (Syahariza et al., 2014; Raigond et al., 2015). The rate and extent of digestibility of starch in the small intestine determine the eventual glucose level in blood, which is of great interest in the context of worldwide health concerns.

Although starch is a homopolysaccharide of glucose, a proportion of starch, namely, resistant starch (RS), is not digested in the small intestine and passed into the large intestine to produce short-chain fatty acids (Englyst and Hudson, 1996; Hu et al., 2016). Due to its digestion resistance, RS is beneficial for inflammatory bowel disease, glycemic index, cholesterol levels, coronary heart disease, and other conditions (Shen et al., 2022). Based on different origins and characteristics, RS can be classified into RSI, RSII, RSIII, RSIV, and RSV five types. RSII consists of native starch granules with a compact structure, majorly exists in raw potatoes, peas, and green bananas (Sajilata et al., 2006), and can become digestible after traditional cooking. RSIII consists of retrograded starches that are principally recrystallized amylose produced after heating starchy food, such as in rice. RSV is a complex of amylose and lipids that improve antidigestion capabilities through forming double helical structures (Hasjim et al., 2010).

Food high in RS is associated with improved cardiovascular health, gut health, and glycemic response, while RS contents in rice and other cereal crops are generally below 3% (Xia et al., 2018; Kumar et al., 2018) and affected by amylose content, starch granule structure, fine structure of amylopectin, and other metabolites such as lipids and sugars (Shen et al., 2022). Apparent amylose content (AAC) and amylopectin with degree of polymerization (DP) 8–12 have been found to be positively related to RS content (Shu et al., 2006; Shu et al., 2007; Shen et al., 2022). RS is decisive in determining the glycemic index of rice (Kumar et al., 2018). Nowadays, several genes involved in starch biosynthesis have been found to be responsible for RS formation in rice grains. The *Wx* gene may regulate RS formation by tuning the amylose content (Itoh et al., 2003; You et al., 2022). *BEIIb* may play an important role in RS formation through modulating the distribution of long and intermediate debranched amylopectin chains (Butardo et al., 2011; Guo et al., 2020; Miura et al., 2021) and tuning other metabolites such as sugar, lipid, and protein contents (Baysal et al., 2020). *be1be2b* double mutant shows higher RS content than the *be2b* mutant (Miura et al., 2021). *SSIIIa* has been verified to be a key gene responsible for the RS content; the loss-of-function of SSIIIa gives rise to a high RS content in rice grain, but the effect of *SSIIIa* on RS content depends partly on the functional *Wx* gene (*Wx^a^*) (Zhou et al., 2016). Double repression of *SSIIa* and *SSIIIa* resulted in higher AAC and RS content (Zhang et al., 2011).

In a preliminary study, we developed a series of rice mutants high in RS (Shu et al., 2007; Shu et al., 2009; Ding et al., 2019). These mutants have significantly lower digestibility (Shu et al., 2009; Zhou et al., 2016) and show great potentialities in lowering the serum glucose level of Type II diabetes patients. However, the absolute content of RS in these mutants showed fluctuations among different growing years or locations (data not published). Bao et al. (2020) also found that the RS content of three chalky mutants derived from R9311 differed significantly between two environments (Hangzhou and Hainan). Among environmental factors, temperature is the most important for rice kernel development. It is proposed that temperature may influence the RS formation and the functional properties of high-RS rice.

With global warming, high temperature (HT) during grain filling has become a great threat for rice production. HT during grain filling can lead to reduced yield, increased chalkiness, and varied amylose content (AC) and starch structure (Zhang et al., 2016). AC has been found either increased or decreased when exposed to HT depending on rice variety (Cheng et al., 2005; Lin et al., 2010; Liu et al., 2021). Zhao et al. (2020) found that *ssIIIa*-RNAi affected the susceptibility of grain chalky occurrence to HT exposure due to the relatively sensitive response of ADP-glucose pyrophosphorylase (AGPase) and SSI to HT exposure and induced significant decreases in AAC and a larger decline in the proportion of DP <12 under HT relative to wild type (WT). Although the formation of RS in rice grain has been investigated in detail from the intrinsic properties of starch, storage and processing conditions, and external factors such as non-starch components (Shen et al., 2022), few concerns have been paid on the growth environment such as temperature. In view of extreme climate change, understanding the influences of temperature on RS formation will provide valuable clues for breeding high-RS rice with improved quality. In the present study, genetic *SSIIIa* mutations in high-RS rice mutants RS111 and RS4 were detected. The main reserve substances in rice under two growth conditions with different day/night temperatures are measured. Furthermore, the expression levels of genes involved in starch synthesis, amylopectin chain length (CL) distribution, X-ray diffraction (XRD) analysis and Fourier transform infrared (FTIR) spectroscopy, and thermal and pasting properties of starch were investigated. In addition, a possible mechanism of temperature affecting RS formation was proposed. The results in this study give some evidence for the impacts of temperature on RS formation in rice grain and provide alternative thoughts on improving the quality of rice high in RS under global climate warming.

## Materials and methods

### Plant materials and growth conditions

Three indica rice varieties with different RS contents were used in this study, including R7954 (WT) and RS111 and RS4 (mutants). RS111 was derived from R7954 through 300 Gy 60-Cobalt γ-ray mutagenesis, and RS4 was obtained from RS111 by irradiation (Shu et al., 2006; Shu et al., 2009); the two mutants have been planted for more than 15 generations. All varieties were grown in artificial climatic chambers with a relative humidity of 80% under a photon flux density of 630–720 μmol·m^-1^·s^-1^. The growth cabinets were maintained at an average air temperature of 25°C (NT, ranging from 20°C to 30°C, 12-h day/12-h night cycles) until flowering. Then, half of the plants were transferred to another growth cabinet with an average air temperature of 30°C (HT, ranging from 25°C to 35°C, 12-h day/12-h night cycles). Individual panicles were labeled on the day of flowering. Grains at 8 days after flowering (DAF) were collected and immediately frozen in liquid nitrogen and stored at -80°C until use. Mature grains were harvested and dried under 37°C, then the glume and aleurone were detached using Satake rice machine (Satake Corp., Japan). After polishing, the white rice was ground into flour, passed through a 150-μm sieve and stored in a drier until use.

### DNA extraction and sequencing

The total genomic DNA of rice leaf was extracted using Tris physiological saline (TPS) (1 M Tris-HCl pH 8, 0.5 M EDTA pH 8, 5 M KCl) according to the procedure modified by Xin et al. (2003). The PCR products amplified with SSIIIa CAPS primer were digested using restriction enzyme TSP509i as previously reported (Zhou et al., 2016) and separated using 2% agarose gel. Genomic *SSIIIa* was amplified using gSSIIIa primer, and the PCR products were sequenced using Genetic Analyzer (3730XL-96, ABI, USA). All primers used are listed in **Table S1**.

### RNA extraction and quantitative real-time PCR

The total RNA from dehulled seeds at 8 DAF was extracted using Plant RNA Extraction Kit (9769, Takara, Japan), and cDNA was synthesized using FastKing RT Kit (KR116, TIANGEN Biotech, China) following the manufacturer’s protocol. Quantitative real-time PCR (qRT-PCR) was performed with SYBR Green Premix Pro Taq HS qPCR Kit (AG11701, Accurate Biotech, China) on the CFX96 Real-time system (Bio-Rad, USA). The rice *Actin* gene (LOC_Os0350885) was used as internal reference. qPCR primers of 27 starch-related genes were referred to Ohdan et al. (2005) and listed in **Table S1**.

### Sample preparation and starch extraction

Starch was isolated using NaOH as follows. The flour and NaOH solution (0.3%, w/v) were mixed (1:20, w/v) thoroughly, shaking at 180 rpm and 4°C for 16 h. Then, the mixture was filtered with a 150-μm sieve, and the filtered solution was centrifuged at 5,000 g for 10 min. The yellow layer in the precipitation was scraped carefully, then the precipitation was suspended with ddH_2_O, and the pH of the slurry was adjusted to neutral. Repeated centrifugation and rinsing with ddH_2_O were done several times until the precipitation was clearly white. Then, the obtained starch was dried at 37°C, ground, and filtered with a 150-μm sieve. The isolated starch was used for XRD, FTIR, differential scanning calorimetry (DSC), and amylopectin chain length (CL) profile analyses.

### Major reserves and RS measurement

AAC and total starch content were measured according to the methods described by Shu et al. (2014). The total protein was measured using Digestor (DT208, FOSS, Denmark) and automatic Kjeldahl nitrogen analyzer (Foss8400, FOSS, Denmark) based on the principle of Kjeldahl method. Lipid content was measured using a fat analyzer (SOX406, Hanon Group, China) based on Soxhlet extraction method.

RS content was measured according to Shu et al. (2014), but the released glucose content was measured using Glucose Assay Kit (Ningbo Saike Biological Technology, China). The glucose content was converted to starch content by a factor of 0.9.

### Amylopectin chain length distribution

Amylopectin was extracted from starch using a fractionation method according to the procedure described by Kong et al. (2008). Purified amylopectin was debranched with pullulanase (E-PULKP, Megazyme, USA) and isoamylase (E-ISAMYHP, Megazyme, USA) using the method described by Kong et al. (2016). The CL distributions of debranched amylopectin were determined using high-performance anion-exchange chromatography (HPAEC) (DionexICS-5000+, Sunnyvale, CA, USA) with BioLC gradient pump and a pulsed amperometric detector (PAD) (Kong et al., 2016).

### X-ray diffraction

The X-ray powder diffractometer (Bruker D8 Advance, Brucker, Germany) was used for XRD analysis. Starch samples were packed into a round glass sample holder and scanned from 4° to 70° 2θ with a step of 0.02°/2 s. The relative crystallinity (RC; %) was calculated using MDI Jade 6.5.

### Fourier transform infrared spectroscopy

Starch granule short-range ordered structure was detected with the FTIR spectrometer (NICOLET iS50FT-IR, Thermo Scientific, USA). The resolution of the instrument is 4 cm^-1^, and the scanning range is from 4,000 cm^-1^ to 400 cm^-1^. The relative absorbances at 1,045, 1,022 and 995 cm^-1^ were extracted from the deconvoluted spectra and were measured from the baseline to the peak height.

### Differential scanning calorimetry

The thermal properties of starch were determined using DSC (Q20, TA Instruments, Newcastle, DE, USA) according to the method described by Shu et al. (2009). The enthalpy change (*ΔH*), onset temperature (To), peak temperature (Tp), and conclusion temperature (Tc) were calculated using Universal Analysis 2000 program (Version 4.4A).

### Rapid viscosity analysis

The pasting properties of rice flours were measured using a rapid visco analyzer (Model 4500, Perten Instruments, Sweden) according to the procedure described by Bao et al. (2004). Peak viscosity (PV), hot paste viscosity (HPV), cold paste viscosity (CPV), breakdown value (BD = PV-HPV), and setback value (SB = CPV-PV) were recorded.

### Statistical analysis

All experiments have biological triplicates. One-way analysis of variance followed by the least significant difference (LSD) test (*P* < 0.05 or *P* < 0.01) was performed using SPSS 22 (IBM, USA).

## Results

### Genotypes of RS111 and RS4

**Figure 1:**
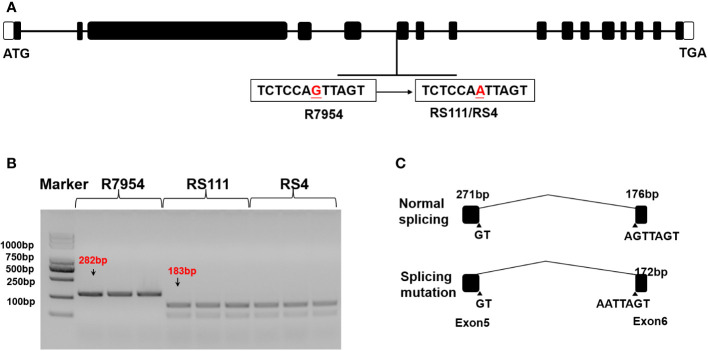
SSIIIa sequence variations in wild type (WT) (R7954) and mutants (RS111 and RS4). **(A)** Genomic sequencing of WT and mutants. **(B)** Genotyping of *SSIIIa* alleles using the *SSIIIa* CAPS marker. **(C)** Varied splicing of transcripts in WT and mutants.

*SSIIIa* has been confirmed to be a key gene for RS formation in rice, as RS111 and RS4 are both derived from R7954 as *b10*, whether they are allelic *ssIIIa* mutations was detected first. Compared with R7954, RS4 and RS111 carried a G/A mutation in the fifth intron of *SSIIIa*, which caused varied splicing and led to a 4-bp deletion in the coding sequence (**Figures 1A, B**). With the SSIIIa CAPS marker developed based on this Single Nucleotide Polymorphism (SNP), the PCR products were digested into two shorter products by TSP509i (**Figure 1C**). This proved that RS4 and RS111 carried the same mutation in *SSIIIa* gene as *b10* (Zhou et al., 2016) and are two allelic *ssIIIa* mutants.

### Main reserve substances in the grains under different temperatures

The main reserves in grains of mutants RS111 and RS4 differed significantly from those in R7954, such as decreased starch content, elevated AAC, RS and lipid contents (**Table 1**). While RS4 and RS111 also showed differences in the contents of the main components, except total starch (**Table 1**). The RS content in RS4 under NT reached up to 7.76% and the lipid content was high up to 2.19%, which was almost two times and three times as high as those in RS111, respectively. In addition, the protein in RS4 was the least but that in RS111 was the highest. Meanwhile, RS4 showed almost transparent grain under NT, although both mutants showed serious chalk under HT (**Figure S1**). It is noteworthy that although both RS4 and RS111 had the same *ssIIIa* mutations, other genes related to starch or lipid metabolites might be mutated in RS4.

**Table 1:** Main components of the three varieties under NT and HT*.

Varieties	Total starch (%)	AAC (%)	RS (%)	Protein (%)	Free lipids (%)
R7954-NT	77.27 ± 1.45a	24.91 ± 0.55c	0.75 ± 0.02e	5.44 ± 0.012c	0.30 ± 0.01e
R7954-HT	73.35 ± 0.66ab	22.74 ± 0.39d	0.79 ± 0.03e	5.31 ± 0.009d	0.91 ± 0.04cd
RS111-NT	67.06 ± 1.07c	27.03 ± 0.14b	3.97 ± 0.04d	5.80 ± 0.01a	0.69 ± 0.04d
RS111-HT	68.82 ± 1.29bc	27.69 ± 0.28b	4.83 ± 0.16c	5.59 ± 0.012b	1.08 ± 0.09c
RS4-NT	67.72 ± 1.26c	32.42 ± 0.10a	7.76 ± 0.2b	4.88 ± 0.005e	2.19 ± 0.07b
RS4-HT	68.81 ± 1.38bc	31.65 ± 0.44a	8.42 ± 0.28a	4.79 ± 0.009f	3.00 ± 0.05a

*The same small letter in the same column indicated that there were no significant differences among different samples at the P < 0.05 level. AAC, apparent amylose content; RS, resistant starch; NT, normal temperature; HT, high temperature.

Under HT, the protein content was decreased in all three varieties and the lipid contents were all increased. The lipid content in R7954 increased up to 0.91%, close to that in RS111, and the lipid content in RS4 reached up to 3.0%. However, the total starch content and AAC in R7954 were reduced by 3.92% and 2.17%, respectively, while those in RS111 and RS4 are similar as those at NT. HT elevated the RS content only in RS111 and RS4 but not in R7954; the RS content increased from 3.97% to 4.83% and from 7.76% to 8.42% in RS111 and RS4, respectively (**Table 1**). This indicated that HT played different influences on the storage accumulations among R7954, RS111, and RS4, although they have the same genetic background.

### Expression pattern of genes involved in starch synthesis

**Figure 2:**
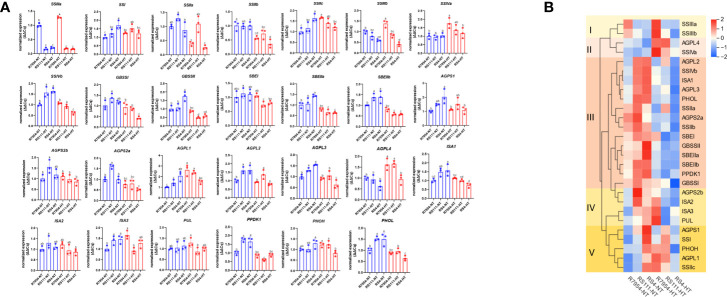
Expression levels of different genes in the three varieties under normal temperature (NT) and high temperature (HT) **(A)** and the clustering according to their expression levels **(B)**. The same small letter indicated that there were no significant differences among different samples at the *P* < 0.05 level.

Among the detected 27 starch synthesis-related genes, the expression of *SSIIIa* was significantly downregulated in both RS111 and RS4, indicating that the mutation of *SSIIIa* diminished its transcript. Compared with R7954, most genes in mutants especially in RS4 were upregulated under NT, while these were downregulated under HT (**Figure 2**). However, the expressions of *SSIIIb*, *AGPL4*, and *SSIIa* were lower in RS4 than those in RS111 and R7954; the expression level of *SSIIa* in RS111 was the highest under both NT and HT (**Figure 2A**).

Upon HT, the expressions of starch-related genes showed different responses among R7954, RS111, and RS4. According to their expressions under NT and HT, all genes tested could be categorized into five groups (**Figure 2B**). *SSIIIa* and *SSIIIb* exhibited higher expressions in R7954 at HT than those at NT but showed similar or even lower expressions in RS111 and RS4 at HT. *AGPL4* and *SSIVa* were upregulated under HT in all three varieties. Group III contains most of the genes, including *SSIIa*, *BEI*, and *SBEIIb*; the expressions of these genes in the three varieties all declined to a certain extent under HT, although the attenuated expression of some genes in R7954 was slight. *ssIIIa* mutation increased the susceptibility of genes, such as *GBSSI*, *SSIVb*, *ISA1*, *BEIIb*, and *BEIIa*, to HT exposure, bringing up larger declines in the expression of most genes detected.

### Amylopectin chain length distributions

**Figure 3:**
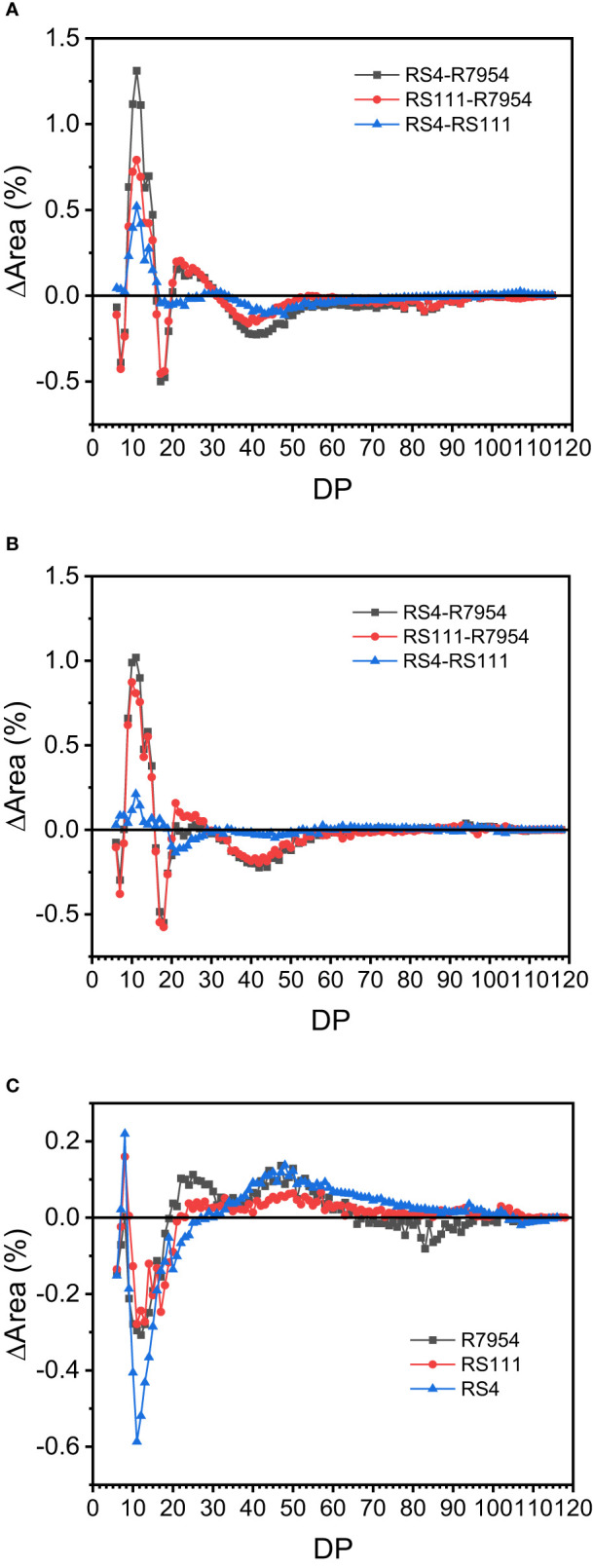
Comparison of the chain length distribution patterns of amylopectin among rice at normal temperature (NT) **(A)**, high temperature (HT) **(B)**, and between HT and NT **(C)**.

Based on the DP, the side chains of amylopectin can be classified into four fractions, namely, fa with DP 6–12, fb1 with DP 13–24, fb2 with DP 25–36, and fb3 with DP ≥37, which correspond to A, B1, B2, and B3 together with longer chains, respectively (Nakamura et al., 1997). The proportions of fa, fb1, fb2, and fb3 from the three varieties under NT and HT were presented in **Table 2**. Among the three varieties, RS4 had the highest proportion of fa and the lowest amount of fb3, followed by RS111 and R7954. High-RS mutants RS111 and RS4 had reduced average chain length of amylopectin (**Table 2**). The varietal differences in amylopectin CL among R7954, RS111, and RS4 were consistent at either NT or HT; mutants had more chains of DP 9–15 and DP 20–30, with the greatest increases in DP 9–12 and less chains of DP ≥37 (**Figures 3A, B**). These results were basically similar to previous results (Fujita et al., 2007; Shu et al., 2007; Zhao et al., 2020). Under HT, the amounts of fa and fb1 were decreased and fb2 and fb3 were increased for all three varieties (**Table 2**), with the greatest decreases in DP 9–19 and increases in DP ≥37 (**Figure 3C**). Obvious decreases in fa under HT were also found in *ssIIIa*-RNAi rice (Zhao et al., 2020). It is noteworthy that the increase or decrease of the proportion of each chain at HT did not correspond to the borders of the fractions. HT resulted in an increase in the proportion of DP 20–30 in R7954 but not in RS111 and RS4 (**Figure 3C**). Several studies also found that HT decreased the proportion of fa and increased the proportions of fb2 and fb3 (Umemoto et al., 1999; Jiang et al., 2003; Zhang et al., 2016). The similar trends of decline in the proportion of chains with DP 9–19 in all three varieties indicated that the mechanism causing the temperature-dependent variation of CL is different from the one specifically regulating the proportions of fa and fb1 caused by the mutation of *ssIIIa*.

**Table 2:** Chain length distribution of amylopectin in all rice materials, as determined by HPAEC-PAD analysis*.

Varieties	fa (6≤DP≤12) (%)	fb1 (13≤DP≤24) (%)	ffb2 (25≤DP≤36) (%)	fb3 (37≤DP) (%)	Peak DP	Average CL	fa/fb1	fa/fb1-2	fa/fb1-3
R7954-NT	24.76 ± 0.17c	48.01 ± 0.33ab	12.53 ± 0.25b	14.7 ± 0.52ab	12	20.77 ± 0.35	0.52	0.41	0.33
R7954-HT	23.4 ± 0.53d	47.22 ± 0.84b	13.28 ± 0.15a	16.1 ± 1.44a	12	22.12 ± 0.21	0.5	0.39	0.31
RS111-NT	26.59 ± 0.17b	48.79 ± 0.16ab	12.79 ± 0.15ab	11.83 ± 0.36cd	12	18.17 ± 0.13	0.54	0.43	0.36
RS111-HT	25.95 ± 0.12b	47.48 ± 0.16ab	13.16 ± 0.14ab	13.41 ± 0.18abc	12	20.73 ± 0.11	0.55	0.43	0.35
RS4-NT	28.25 ± 0.2a	49.14 ± 0.16a	12.76 ± 0.11ab	9.84 ± 0.31d	12	18.26 ± 0.29	0.57	0.46	0.39
RS4-HT	26.66 ± 0.07b	47.19 ± 0.03b	12.91 ± 0.04ab	13.24 ± 0.07bc	12	20.18 ± 0.21	0.56	0.44	0.36

*The same small letter in the same column indicated that there were no significant differences among different samples at the P < 0.05 level. CL, chain length.

### Long-range and short-range ordered structure

**Figure 4:**
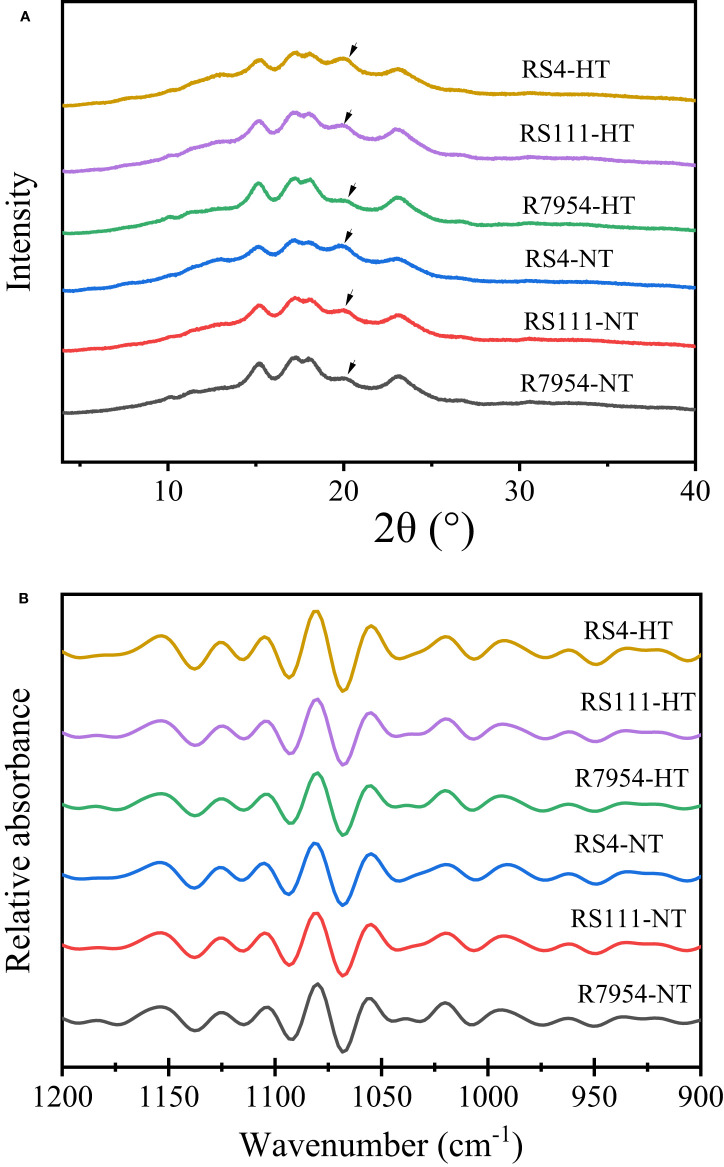
X-ray diffraction (XRD) spectra **(A)** and deconvoluted Fourier transform infrared (FTIR) spectroscopy spectra **(B)** of starches from wild type (WT) and mutants at normal temperature (NT) and high temperature (HT). The arrow in panel **(A)** indicated the peaks of starch–lipid complexes.

XRD can be used to measure the helices that are packed in regular arrays (in the long-range distance) forming crystallinity (Man et al., 2012). The XRD pattern of all starches showed diffraction peaks at ca. 15°, 23° and an unresolved doublet at ca. 17° and 18° (**Figure 4A**), which are the typical A-type crystalline pattern (Pérez and Bertoft, 2010). In addition, all XRD patterns showed a diffraction peak around 2θ 20°, which indicated the existence of the amylose–lipid complex (Putseys et al., 2010). RS4 had the lowest RC, but the most starch–lipid complexes, especially under HT (**Table 3**). This might be due to the higher lipid content and AAC in RS4 and RS111 (**Table 1**). Under HT, RC and the starch–lipid complexes of RS111 and RS4 were elevated but not for R7954 (**Table 3**).

**Table 3:** RC and IR ratios of starches from R7954, RS111 and RS4 at NT and HT*.

Sample	RC (%)	Starch–lipid complexes (%)	IR ratio	IR ratio
			1,045/1,022 (cm^-1^)	1,022/995 (cm^-1^)
R7954-NT	30.62 ± 0.15a	1.05 ± 0.01a	0.648 ± 0.0004e	1.137 ± 0.0021a
R7954-HT	30.13 ± 0.31a	1.10 ± 0.01a	0.664 ± 0.0004d	1.106 ± 0.0016b
RS111-NT	27.82 ± 0.26b	1.66 ± 001b	0.668 ± 0.0007c	1.058 ± 0.001e
RS111-HT	30.34 ± 0.11a	1.94 ± 0.03c	0.663 ± 0.0003d	1.099 ± 0.0006c
RS4-NT	25.42 ± 0.11c	2.46 ± 0.05d	0.68 ± 0.0005a	1.04 ± 0.0008f
RS4-HT	27.11 ± 0.09b	2.76 ± 0.04e	0.674 ± 0.0009b	1.078 ± 0.0014d

*The same small letter in the same column indicated that there were no significant differences among different samples at the P < 0.05 level.

RC, relative crystallinity; NT, normal temperature; HT, high temperature.

With FTIR, short-range ordered structure of starch granule can be observed, the ratio of absorption at 1,045/1,022 cm^-1^ and 1,022/995 cm^-1^ can reflect the degree of order structure and the proportion of amorphous to ordered carbohydrate structure in starch granule external region, respectively (Sevenou et al., 2002). All three varieties showed similar FTIR profiles under both NT and HT; no new peaks appeared (**Figure 4B**). RS111 and RS4 showed higher 1,045/1,022 cm^-1^ ratio but lower 1,022/995 cm^-1^ ratio under NT (**Table 3**), indicating that there were more short-range order helical structure in the external region and less proportion of amorphous to ordered structure in the mutants. Under HT, the 1,045/1,022 cm^-1^ ratio of R7954 was elevated but that of RS111 and RS4 lessened (**Table 3**), indicating that the short-range ordered structure especially in the external region of the starch granule in RS111 and RS4 was slightly attenuated. HT played more apparent influences on starch ordered structure in mutants than in R7954, especially on long-range ordered structure.

### Thermal properties and pasting properties

At NT, RS111 and RS4 had lower To, Tp, and *ΔH* than R7954. Compared with the values of To (58.91°C–61.02°C), Tp (63.33°C–65.52°C), Tc (69.55°C–74.29°C), and *ΔH* (5.68–8.65 J/g) at NT, it is obvious that HT significantly elevated the gelatinization temperature (GT) and *ΔH* (**Table 4**).

**Table 4:** Thermal properties of R7954, RS111, and RS4 at NT and HT*.

Varieties	T_o_ (°C)	T_p_ (°C)	T_c_ (°C)	*ΔH* (J/g)
R7954-NT	61.02 ± 0.08c	65.52 ± 0.03c	71.13 ± 0.11d	8.65 ± 0.07b
R7954-HT	63.25 ± 0.13a	69.06 ± 0.05b	75.72 ± 0.14b	10.46 ± 0.04a
RS111-NT	58.89 ± 0.04e	63.33 ± 0.05e	69.55 ± 0.22e	7.07 ± 0.01d
RS111-HT	60.16 ± 0.04d	65.12 ± 0.04d	71.9 ± 0.39d	8.46 ± 0.03c
RS4-NT	58.91 ± 0.1e	65.09 ± 0.55d	74.29 ± 1.25c	5.68 ± 0.4e
RS4-HT	61.68 ± 0.01b	69.89 ± 0.21a	79.53 ± 0.36a	7.2 ± 0.05d

*The same small letter in the same column indicated that there were no significant differences among different samples at the P < 0.05 level.

As for the paste viscosity, RS111 and RS4 showed significantly lower paste viscosity than R7954 regardless of whether at NT or HT (**Table 5**). The PV of R7954, RS111, and RS4 at NT was 1,787 cP, 277 cP, and 200.5 cP, respectively. BD and SB of RS111 and RS4 were also lower than those of R7954. Other *ssIIIa* mutants also showed very low viscosity (Fujita et al., 2007; Zhang et al., 2011). Under HT, the PV, HPV, CPV, and SB values were decreased in all three varieties, indicating that HT contributed greatly to pasting properties of rice starches, and the cooking quality of rice grain decreased under HT. Decreased paste viscosity of rice under HT had also been observed by Zhang et al. (2020).

**Table 5:** Viscosity parameters of R7954, RS111, and RS4 under NT and HT*.

Varieties	PV (cP)	HPV (cP)	CPV (cP)	BD (cP)	SB (cP)
R7954-NT	1787 ± 12.26a	1658 ± 20.27a	2680 ± 39.13a	129 ± 8.01b	893 ± 26.87a
R7954-HT	1701 ± 26.4b	1496 ± 16.5b	2213 ± 0.94b	205 ± 9.9a	512 ± 27.34b
RS111-NT	277 ± 7.54c	269 ± 5.66c	413.5 ± 14.85c	8 ± 1.89c	136.5 ± 7.31c
RS111-HT	241 ± 1.41dcd	239 ± 1.89cd	314.5 ± 0.24d	2 ± 0.47c	73.5 ± 1.65cd
RS4-NT	220.5 ± 2.12d	217.5 ± 2.12d	255.5 ± 2.59de	3.33 ± 0.27c	35 ± 0.47de
RS4-HT	204.5 ± 2.59d	200.5 ± 2.59d	209 ± 2.36e	4.33 ± 0.27c	4.5 ± 0.24e

*The same small letter in the same column indicated that there were no significant differences among different samples at the P < 0.05 level. PV, peak viscosity; HPV, hot paste viscosity; CPV, cold paste viscosity; BD, breakdown value; SB, setback value.

## Discussion

### The influences of ssIIIa mutation and high temperature on RS and other accumulations in rice grains

The *SSIIIa* gene has been identified as the key gene for RS formation in rice grains (Zhou et al., 2016). RS111 and RS4 are two *ssIIIa* allelic mutants, and the increased AAC and RS content in RS111 and RS4 (**Table 1**) might be majorly due to the mutation of *SSIIIa* (**Figure 1**). However, RS4 had higher RS and lipid contents (**Table 1**) that contribute to its greatest starch–lipid complexes (**Table 3**), as AAC can complex with lipids to form RSV (Hasjim et al., 2010; Zhou et al., 2016). This indicated that, besides *SSIIIa*, other genes involved in starch synthesis or lipid metabolites might also contribute to RS formation in rice grains.

During grain development, an increment in temperature of 1.6°C–3.1°C can disturb the accumulation of storage materials (Liu et al., 2021). Previous studies showed that HT can increase protein content (Lin et al., 2010) and was unfavorable for lipid accumulation during rice grain ripening. However, HT showed negative influences on protein content and stimulated lipid accumulation in this study (**Table 1**), although the decreased protein content is small in absolute values. This might be due to different varieties studied. Variations in AAC under HT may also be dependent on rice varieties. Cheng et al. (2005) found that HT increased the AC in high-AC varieties, while Liu et al. (2021) found that AC decreased under elevated temperatures. The different responses of AAC in R7954 and mutants RS111 and RS4 to HT (**Table 1**) might be because of the increased sensitivity of amyloplast enzymes in RS111 and RS4 exposed to HT. AAC has been thought to be a key determinant for RS formation; higher AAC always related to higher RS content (Raigond et al., 2015). The inconsistent decreases or increases in AAC and RS in the three varieties under HT indicated that, besides amylose, other factors such as lipids also play important roles in RS formation under HT.

### Impacts of *ssIIIa* mutation and high temperature on the expressions of genes involved in starch synthesis and amylopectin fine structure

SSIIIa is a key enzyme responsible for the elongation of long branched amylopectin; it might regulate the whole cascade of starch biosynthesis through the formation of large enzyme complexes with other enzymes (Crofts et al., 2012; Crofts et al., 2015). Loss-of-function mutation of *SSIIIa* results in abnormal starch metabolites in RS111 and RS4, resulting in a lower total starch content, higher AAC and lipid content, and more proportion of DP 9–15 and DP 20–30 when compared with those of R7954 (**Figure 3**). The enhanced expression of *SSIIa* in RS111 might partly compensate for the loss-of-function of *SSIIIa*, as they play partially overlapping roles during starch synthesis (Zhang et al., 2011), which might partially explain the differences in CL distribution of amylopectin between RS111 and RS4 (**Figure 2**).

Generally, HT leads to a declined expression of many starch biosynthesis genes such as *GBSSI*, *BEs*, *cyPPDK* (Jiang et al., 2003; Yamakawa et al., 2007; Zhang et al., 2021) and *SSs* like *SSIIIa*, *SSII*, and *SSIV* (Liu et al., 2021). SSI, SSIIa, SSIIIa, BEI, and BEIIb function specifically in elongating or synthesizing A chains, B1 chains, B2 chains, or longer chains (Nakamura et al., 2005; Fujita et al., 2006; Fujita et al., 2007; Jeon et al., 2010). The decreased transcripts of starch synthase and branching enzyme genes (**Figure 2**) in all varieties resulted in decreases in the amount of fa and fb1 under HT (**Table 2**, **Figure 3**) (Jiang et al., 2003). Meanwhile, lower expression levels of *SSI*, *SSIIc*, and some other genes in RS4 under HT (**Figure 2**) might be responsible for its noticeable decreases in amylopectin short chains. The more pronounced decreases in the expression of several genes such as *BEIIb*, *BEI* and *BEIIa* in RS111 and RS4 indicated that *ssIIIa* mutation exacerbated the influences of HT on starch metabolites.

### Influences of high temperature on starch crystalline

Crystallinity was influenced majorly by fine amylopectin structure and AC (Man et al., 2014; Bertoft, 2017). More AAC and shorter amylopectin usually lead to a defective crystallite (Luo et al., 2021). The lower RC in RS111 and RS4 might be due to their higher AAC (**Table 1**) and more short chains of amylopectin (**Table 2**). The increased RC in RS4 and RS111 at HT was consistent with the pronouncedly decreased amount of fa and fb1 and increased proportion of fb2 and fb3 at HT (**Table 2**). Although amylopectin long chains are of benefit for the formation of double helices, complexing with non-starchy compounds increased short-range ordered structure (Chi et al., 2021). The increased starch–lipid complexes in RS111 and RS4 might contribute to the increased 1,045/1,022 cm^-1^ ratio (**Table 3**).

Previous studies indicated that slower digestibility of processed starch is usually related to the short-range ordered structures; however, the reassembled ordered molecular aggregation architecture showed more pronounced starch digestibility. Compared with long-range ordered structures, short-range ordered structures exhibited a weaker effect on starch digestibility (Chi et al., 2021). The increased RC in RS4 and RS111 under HT might partly contribute to their enhanced RS content, as an increase in the degree of crystallinity can result in a reduction in starch digestibility *in vitro* and *in vivo* (Rombo et al., 2001; García-Rosas et al., 2009), and the amorphous matrix might be more susceptible to enzyme attack (Chung et al., 2006).

### Influences of high temperature on starch thermal and paste viscosity

GT has been found to be related to crystallinity associated with molecular order (**Table 3**). The amount of short-chain amylopectin (DP ≤12) was negatively correlated with GT; starch with relatively high levels of long branch chains (13 ≤ DP ≤ 24) requires higher temperatures for complete dissociation (Zhang et al., 2020). The higher AAC (**Table 1**) and the more proportion of fa in mutants (**Table 2**, **Figure 3**) indicated that they have shorter double helices formed between adjacent chains in the crystalline lamellae (Jeon et al., 2010), leading to a lower starch GT and *ΔH* (**Table 4**). The enhanced GT under HT may be due to the increased crystallites and double helices (**Table 3**). Under HT, the increased proportion of amylopectin long chains that span at least two crystalline lamellae and more ordered structures can form more stable double helices; hence, a higher GT would be required to disorder the ordered structure (Witt et al., 2012). A previous study also found that HT exposure led to a significantly higher GT (Pang et al., 2021). Furthermore, the variations of GT between NT and HT were larger than those among varieties (**Table 4**), indicating that the influences of temperature on GT were more obvious than that of *ssIIIa* mutation.

The very low viscosity of RS111 and RS4 might be due to their high AAC and lipids (**Table 1**), as lipids and amylose complexes formed during cooking can render entanglements with amylopectin molecules and restrict the swelling of granules, causing incomplete gelatinization during cooking (Sun et al., 2018), which results in a higher pasting temperature and a lower PV (Ai and Jane, 2015). Increased lipids under HT (**Table 1**) might be responsible partially for the decreased viscosity. Previous studies have found that *Wx* and *SSIIa* were the main genes involved in many grain quality properties such as gelatinization and paste viscosity (Kharabian-Masouleh et al., 2012); significantly different expressions of *GBSSI* and *SSIIa* among R7954, RS4, and RS111 at both NT and HT might explain the different viscosity among them.

### Proposed regulations of high temperature on RS formation in rice

Rice high in RS had a relatively higher proportion of DP 8–12 and less DP ≥37 (Shu et al., 2007). Among the three varieties, the higher the RS content, the most the proportion of DP 9–15 and the least the proportion of DP ≥37. While under HT, the amylopectin chains with DP 9–15 in RS4 and RS111 were greatly decreased and chains with DP ≥37 were increased, but the RS contents in RS111 and RS4 under HT were significantly increased (**Table 1**). The incongruent correlation between RS and DP 9–15 and DP ≥37 under HT indicated that the mechanism causing enhanced RS under HT should be different from that caused by loss of function of *SSIIIa*, which might be due to the differences in stability or temperature preference of starch synthase and starch branching enzyme (Umemoto et al., 1999).

**Figure 5:**
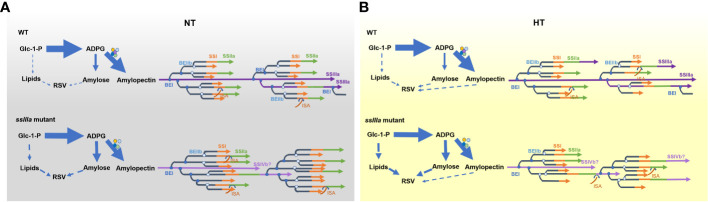
Proposed processes of RS formation in rice. **(A)** The loss-of-function mutation of SSIIIa causes repartition of carbohydrate, breaks the equibrium synthesis of amylose and amylopectin, and leads to higher AAC and lipid content that promote the formation of starch–lipid complexes. **(B)** Loss-of-function mutation of SSIIIa leads to more short chains of DP 9–15 both under NT and high temperature (HT). HT decreases the branching, resulting in less proportion of short chains and more long chains. The increased long chains and enhanced lipid content under HT stimulate more RS accumulation.

The synthesis of amylose and amylopectin might compete for substrates and enzymes (Han et al., 2019) and maintain equilibrium under normal conditions. While inactivation of *SSIIIa* results in varied transcripts of genes related to starch synthesis (**Figure 2**), leading to more substrates synthetized by GBSSI into amylose (**Figure 5**). Furthermore, inactive SSIIIa facilitated the synthesis of DP 10–15 chains from short DP 6–9 chains of A chains and DP 20–30 chains from DP 16–19 chains of B1 chains of amylopectin by enhancing the endogenous SSI activity and reduced the formation of DP >35 chains (Fujita et al., 2007) (**Table 2**, **Figure 5B**). Enhanced transcripts of SSIVb or other enzymes might partially compensate for the elongation of B2-B4 in the *ssIIIa* mutant (**Figure 5**). Concurrently, the increased expression levels of *BEs* such as *BEI* and *BEIIb* (**Figure 2**) might increase the branching frequency and enhance the proportion of short chains further (**Table 2**, **Figure 5**). Less proportion of short-chain DP 10–15 is an important determinant for high RS (**Table 1**) (Shu et al., 2007; Shu et al., 2009). Additionally, pyruvate phosphate dikinase (PPDK) presented in the complex with AGPase may enable G-1-P channel to lipid, while the starch biosynthetic complexes are proposed to inhibit the activity of PPDK and AGPase, the absence of SSIIIa enhanced the expression of *PPDK* (**Figure 2**) and promoted lipid accumulation (**Figure 5**). The high levels of amylose and lipids in mutants especially in RS4 can form more amylose–lipid complexes (RSV) (**Table 3**).

Under HT, the altered transcripts of several genes in R7954 led to aberrant starch synthesis (**Table 1**). Fan et al. (2019) found that the ratio of SBE activity to that of GBSS was increased under HT. Downregulation of the *Wx* gene due to transcript inhibition or posttranscriptional regulation (Zhang et al., 2021) caused less synthesis of amylose, while for *ssIIIa* mutants, although the expression levels of most genes were also reduced, amylopectin and amylose biosynthesis maintained a relative balance, but the starch enzyme complexes might be disrupted further due to the lower activity of most amyloplast enzymes and more carbon were partitioned into lipid metabolites, leading to the synthesis of more lipids (**Table 1**, **Figure 5**). Furthermore, decreases in the transcripts of *BEI* and *BEIIb* (**Figure 2**) resulted in lower branching frequency (**Figure 5**) and produced fewer short chains of DP ≤19, with the greatest decreases in chains of DP 9–19 (**Figure 3**). The increased transcripts of *SSIIIa* in R7954 and *SSIVb* in mutants under HT (**Figure 2**) might also partially contribute to the increased proportion of long chains (DP >37) (**Figure 3**, **Table 2**), reflected as intensified crystallinity in mutants (**Table 3**). Longer amylopectin side chains might also be involved in RS formation, although the short chains of DP 9–15 are the major determinants for RS properties, as retrograded amylopectin may also reduce enzyme susceptibility (Eerlingen and Delcour, 1995). Moreover, aside from amylose, long chains of amylopectin may also complex with the increased free lipids and lead to increases in the contents of RSV at HT (**Table 3**). The molecular mechanisms of RS produced in high-RS rice mutants under HT might not be uniform with that under NT. Trimming the fine structure of amylopectin and regulating lipid metabolites through biological means might be alternative ways to obtain rice rich in RS with acceptable palatability under global climate change.

In conclusion, RS4 and RS111 are two *ssIIIa* allelic mutants as *b10* (Zhou et al., 2016). Meanwhile, other mutations responsible for RS formation might exist in RS4, as the RS in RS4 was significantly higher than that in RS111. Loss-of-function mutation of *SSIIIa* boosted the expressions of most genes involved in starch biosynthesis under NT, such as *Phol*, *SSI*, *BEIIb*, and *SSIVb*. The varied expressions of genes involved in starch biosynthesis in *ssIIIa* mutants caused the increases in AAC and lipid content, more proportion of DP 9–15, and less proportion of DP >37, which finally led to enhanced RS. However, most of the genes upregulated under NT in *ssIIIa* mutants especially in RS4 were downregulated under HT, such as *GBSSI*, *SSI*, *BEI*, *BEIIb*, and *ISA*. The decreased activities of SSs and BEs under HT caused greatest decreases in chains of DP 9–19 and produced more proportion of long chains (DP >37) in all materials, but only reduced AAC and total starch content in R7954. Moreover, the varied transcripts of genes involved in starch biosynthesis under HT channeled more carbon flux to lipid synthesis, promoting lipid accumulation. The varied starch structure and lipid content under HT resulted in increased crystallinity, GT, *ΔH* and reduced viscosity. The high AAC and enhanced long amylopectin chains and lipid content in RS111 and RS4 under HT might boost the starch–lipid complexes further and contribute to the elevated RS.

## Data availability statement

The original contributions presented in the study are included in the article/**Supplementary Material**. Further inquiries can be directed to the corresponding author.

## Author contributions

YZ: Investigation, Data curation, Formal analysis, Writing-original draft. ZC: Investigation, Data curation, Formal analysis, Writing-original draft. SJ: Investigation. JC: Investigation. DW: Funding acquisition, material bred. XS: Conceptualization, Formal analysis, Writing-review and editing, Supervision and Funding acquisition. All authors contributed to the article and approved the submitted version.

## Funding

This project was financially supported by grants from the National Key Research and Development Program of China (2021YFF100020), Functional Rice Breeding and germplasm enhancement (2022C02011, 2021C02063), National Natural Science Foundation of China (32072038) and Administration Bureau of Yazhou Bay Science Technology City (SKJC-2020-02-010).

## Conflict of interest

The authors declare that the research was conducted in the absence of any commercial or financial relationships that could be construed as a potential conflict of interest.

## Publisher’s note

All claims expressed in this article are solely those of the authors and do not necessarily represent those of their affiliated organizations, or those of the publisher, the editors and the reviewers. Any product that may be evaluated in this article, or claim that may be made by its manufacturer, is not guaranteed or endorsed by the publisher.
