# Supplementary material for: High temperature boosts resistant starch content by altering starch structure and lipid content in rice ssIIIa mutants
Source: Front Plant Sci. 2022 Nov 18;13:1059749. doi: 10.3389/fpls.2022.1059749 (PMC9715984; doi:10.3389/fpls.2022.1059749)
Supplement: Supplementary file 1 [file DataSheet_1.docx]

Figure 1S The grain morphology of three varieties under NT and HT
